# CONFIDENCE 2.0: Preliminary Findings of a Feasibility Study of a Refined, Digitally‐Enhanced Intervention for Latino Caregivers

**DOI:** 10.1002/alz70858_097848

**Published:** 2025-12-24

**Authors:** Kylie Meyer

**Affiliations:** ^1^ Case Western, Cleveland, OH, USA

## Abstract

**Background:**

Latino family caregivers to persons living with Alzheimer's disease and related dementia (AD/ADRD) experience higher levels of financial strain than non‐Latino caregivers. CONFIDENCE is a culturally tailored psychoeducational intervention designed to reduce financial strain by building caregiver resourcefulness to navigate complex resource environments to displace caregiving costs. An initial version of CONFIDENCE demonstrated preliminary efficacy at lowering financial strain, but attendance in facilitated videoconference sessions was modest (3.13 of 5 sessions). The intervention was refined to integrate a web‐based app to support engagement. This study aims to evaluate the preliminary efficacy and feasibility of the refined, digitally enhanced intervention.

**Method:**

This study used a single‐arm pre‐ and post‐test feasibility study design to test the feasibility of the revised CONFIDENCE intervention. Caregivers completed self‐administered online surveys before the intervention, immediately post‐intervention, and two months post‐intervention. Surveys asked about resourcefulness, level of financial toxicity, and the perceived value of the intervention and usability of the app. The intervention included attending four weekly videoconference sessions, completing practice activities between sessions, and registering for a web‐based app that provided text message reminders between lessons, an AI digital assistant to answer questions, and digital access to the intervention workbook and activities. Analyses included descriptive statistics and paired t‐tests.

**Result:**

From April 2024 to October 2024, twelve caregivers enrolled in the revised CONFIDENCE intervention. Enrolled caregivers attended, on average, 2.25 of 4 sessions (SD=1.6). Preliminary findings indicate lower levels of financial strain two months post‐intervention relative to baseline (*p* <0.05) and a trend towards increased resourcefulness (*p* <0.10). Caregivers reported overall satisfaction with the refined intervention, with 7 of 10 indicating participating was worth their time. Engagement in the app was low. The average system usability score for the app was 60.9 (SD=29.2), a “marginally acceptable” score.

**Conclusion:**

Preliminary findings from the digitally enhanced CONFIDENCE intervention demonstrate consistent findings regarding preliminary efficacy but little change in attendance. The app's perceived usability may have diminished anticipated benefits. The investigators are exploring enhanced technology support to improve usability. Continued research is needed to examine how to incorporate digital tools into caregiver psychoeducational interventions.

